# Rethinking and transforming health systems for dementia care in low- and middle-income country settings

**DOI:** 10.1371/journal.pgph.0005419

**Published:** 2025-12-03

**Authors:** J. Jaime Miranda, Miriam Lúcar-Flores, Antonio Bernabe-Ortiz, Rafael A. Calvo, Maria Kathia Cardenas, Nilton Custodio, Yuri L. Cutipé-Cárdenas, Francisco Diez-Canseco, Jemma Hawkins, Filipa Landeiro, Maria Lazo-Porras, Graham Moore, Jose Carlos Vera-Tudela, William Whiteley, Maria Sofia Cuba, Christopher R. Butler, IMPACT Salud Research Group

**Affiliations:** 1 CRONICAS Centre of Excellence in Chronic Diseases, Universidad Peruana Cayetano Heredia, Lima, Peru; 2 Sydney School of Public Health, Faculty of Medicine and Health, The University of Sydney, New South Wales, Australia; 3 Nuffield Department of Population Health, Health Economics Research Centre, University of Oxford, Oxford, United Kingdom; 4 Dyson School of Design Engineering, Imperial College London, London, United Kingdom; 5 Instituto Peruano de Neurociencias, Lima, Peru; 6 Instituto Nacional de Salud Mental Honorio Delgado Hideyo Noguchi, Lima, Peru; 7 Centre for Development, Evaluation, Complexity and Implementation in Public Health Improvement (DECIPHer), School of Social Sciences, Cardiff University, Cardiff, United Kingdom; 8 Centre for Clinical Brain Sciences, The University of Edinburgh, Edinburgh, United Kingdom; 9 Centro de Investigación en Atención Primaria de Salud, Facultad de Medicina, Universidad Peruana Cayetano Heredia, Lima, Peru; 10 The George Institute for Global Health, School of Public Health, Imperial College London, London, United Kingdom; 11 Department of Brain Sciences, Imperial College London, London, United Kingdom; PLOS: Public Library of Science, UNITED STATES OF AMERICA

Increased life expectancy in low- and middle-income countries (LMICs) is driving a dramatic rise in the number of people living with chronic conditions, including dementia. Globally, an estimated 57.4 million people are living with dementia. By 2050, this number is projected to rise to 152.8 million, with the sharpest increases in LMICs [1].

Dementia presents unique challenges due to the complexity of its diagnosis and management, challenges which are compounded by limited resources within health systems in LMICs. The burden of caring for individuals with dementia extends beyond the healthcare sector and involves families and society at large. This is especially pronounced in contexts where health services are fragmented, and social protection is inadequate [2].

There is a crucial need to rethink and transform dementia care in LMICs. In this article, we discuss some of the key challenges and highlight emerging opportunities for sustainable, equitable, and innovative progress. We will use Peru as an illustrative context, given its recent efforts to develop a comprehensive dementia response by establishing a dedicated legal framework and working towards its implementation, as well as integrating digital technologies and community-driven models within its constrained, fragmented health system.

## LMICs deserve better dementia care, but there are challenges

Rethinking dementia care in LMICs is essential not only because of increasing prevalence and finite resources but also to avoid replicating the mistakes and limitations of healthcare delivery models in high-income countries (HICs). Current dementia pathways in HICs face numerous challenges, including staffing shortages, funding constraints, and an emphasis on clinical activities that require substantial financial, service, infrastructure, and specialised human resources. Such models of healthcare will likely not be suitable to be reproduced in LMICs due to their very different political, socioeconomic and healthcare structures. A different approach is proposed, in which models are designed and/or translated to align with the needs and context of LMICs.

### The complex and costly nature of dementia care to health systems

The diagnosis of dementia is complex and costly. Dementia is not a specific disease but a clinical syndrome resulting from various, typically degenerative, brain disorders that cause cognitive dysfunction and vulnerability by impairing daily activities and independent function [3]. In the early stages of dementia, reaching a diagnosis can be difficult and require monitoring change over time. The approach to diagnosing and treating dementia in HICs typically relies on highly specialised services, such as psychiatry, behavioural neurology, brain imaging and neuropsychology, which come at considerable costs to the health system. These services are scarce in LMICs, where relevant infrastructure, specialised training, and healthcare access are unevenly distributed or do not exist [4]. Diagnosis is further complicated by lack of awareness and understanding of what dementia is, leading many to misinterpret its symptoms as part of normal ageing [3].

Care within the health system is also challenged by the cost and complexity of dementia management, especially when it coexists with other long-term conditions. The disability and care needs of people with dementia increase and vary over time. Moreover, dementia is strongly associated with multimorbidity: on average, people living with dementia have twice as many physical and mental health conditions as those without dementia [5,6]. Dementia shares risk factors with many other diseases, including unhealthy behaviours such as poor diet, smoking, and physical inactivity; as well as conditions like obesity, high blood pressure, diabetes, depression, hearing loss and social isolation. Collectively, these potentially modifiable risk factors explain 45% or more of dementia cases globally, a figure that rises to around 54% in Latin American countries, where social and economic inequalities exacerbate these risks [7,8]. A recent study in Latin American populations demonstrated that factors related to social and health disparities—such as mental health symptoms, social determinants of health, education, physical activity, and cardiometabolic factors—are stronger predictors of cognitive and functional decline than classical risk factors like age and sex [9]. Whilst we acknowledge that more research is required to establish causal links between dementia and some of these associated factors, it is clear that preventing and managing dementia requires addressing not just one risk factor or disease in isolation but rather a constellation of intertwined challenges.

Given the complexity of dementia and its rapidly rising prevalence, the societal and economic burdens are substantial. According to certain measurements, the global cost of this condition is predicted to increase from US $2.8 trillion in 2019 to US $16.9 trillion in 2050, with LMICs expected to shoulder 66% of the economic burden by 2050 [10]. These forecasts underscore the urgent need for integrated care pathways for dementia, pathways that require collaboration between multiple actors. This can be challenging in HICs, but the difficulties LMICs face due to poor integration between healthcare providers and systems are far more pronounced. The fragmented structures of health systems in LMICs, combined with limited trained human resources, create infrastructural constraints, and limited diagnostic and treatment capacity, making it hard for clinicians from different specialities and other relevant stakeholders to collaborate effectively in dementia care and hindering the introduction of comprehensive and sustainable change [11,12].

### The broader impact of dementia on families and communities

Beyond the health system, dementia impacts multiple aspects of individual and family well-being and society at large. Limited access to information about dementia, as well as to primary and specialist care services, exacerbates the challenges of living with dementia, particularly in LMICs [7,9]. In many resource-constrained settings, progressive cognitive decline and behavioural changes related to dementia are considered part of normal ageing or mistakenly diagnosed as mental illnesses such as depression [13]. For instance, the 2024 World Alzheimer Report found that in LMICs 68% of health and care professionals think that “dementia is a normal part of ageing” [13]. Also, individuals with dementia may encounter stigma and discrimination, leading to further delays in diagnosis and access to appropriate care. The same report found that in LMICs 32% of the general public agree with the statement that “people with dementia are dangerous more often than not” [13].

Cultural beliefs and practices significantly influence how dementia is perceived and managed in different communities. In some cultures, dementia symptoms may be attributed to supernatural causes or seen as a natural part of ageing, which can delay seeking medical help to prevent further deterioration [14]. Understanding and respecting these cultural perspectives is crucial for developing effective public health strategies and interventions [15].

Support for people living with dementia in LMICs primarily comes from family members, especially women, who provide the bulk of unpaid caregiving [16].This informal care represents a substantial and largely unaccounted-for proportion of dementia care costs, including lost productivity [17]. In LMICs, where formal care workers and infrastructure are limited, these indirect costs have significant social and economic consequences: family caregivers face a higher risk of anxiety, depression, and a lower quality of life compared to caregivers of individuals with other conditions, and households can often be pushed into poverty [16,18,19]. Despite their lack of adequate basic training, caregivers contribute to relieving the burden on the healthcare system by providing essential care and support in daily living activities of people with dementia.

Dementia also affects and requires adaptations in the wider society. As argued by the Social Model of Disability, while any person experiencing impairment may face particular restrictions, disability is created by a cultural, social and environmental barriers that prevent inclusion [20]. Accordingly, there is an urgent need to develop dementia-friendly communities that can reduce isolation, promote social engagement, and adapt environments to meet the needs of people living with dementia as well as provide support and relief to their carers [21]. Such communities can play a key role in reducing stigma, fostering dignity, and supporting the autonomy of people living with dementia.

## Emerging opportunities

Dementia research is advancing rapidly, offering new possibilities. Regarding dementia prevention, research has identified 14 preventable risk factors that might prevent or delay nearly half of dementia cases [22]. There is also progress in both diagnosis and treatment. However, the accessibility and applicability of these innovations often remain concentrated in high-resource settings, raising challenges for equitable care delivery worldwide. In this section, we explore the latest breakthroughs in diagnostic tools, pharmacological and non-pharmacological treatments, and the role of care systems. We also highlight efforts to improve representativeness in dementia research and discuss how policy initiatives can better address the growing global burden of dementia. These developments open new pathways for enhancing dementia care and research, particularly in LMICs.

There has recently been considerable progress in diagnostic techniques and disease-modifying therapies for dementia. Digital tools for cognitive assessment and blood-based biomarkers for neurodegenerative diseases have made significant strides toward more timely, reliable, and scalable diagnosis [22,23], and offer potential solutions to the currently low rates of dementia detection in LMICs if effective diagnostic tools can be placed in the hands of primary care physicians and other non-specialist healthcare professionals [24,25]. Nevertheless, validation of these tools in relevant LMIC populations remains pending [26,27]. Recent pharmacological treatments provide some optimism that the course of Alzheimer’s disease can be altered [28,29]. However, while these treatments offer hope, their clinical effects are relatively modest, and the costs remain prohibitively high for many. Non-pharmacological strategies therefore continue to play a crucial role in managing cognitive, emotional, and behavioural symptoms, helping to preserve functionality and independence for individuals living with dementia [30,31].

Alongside these approaches care navigation systems such as the Care Ecosystem model, which provides continuous support to individuals with dementia and their caregivers through dedicated care team navigators, are emerging as effective ways to provide personalised and cost-effective care [32,33]. With the support of the navigators, these models help patients and families bridge gaps between them and the health system by coordinating care, connecting them with resources, and addressing their ongoing needs in a more personalised, holistic manner. Community-based interventions, such as cognitive stimulation therapy and local support networks, are also providing evidence-based care by engaging people with dementia in activities that enhance cognitive function and social interaction while simultaneously providing caregivers with much-needed support [34]. For instance, iSupport, an online programme for dementia caregivers developed by the World Health Organisation (WHO), provides accessible information, strengthens caregivers skills and has been adapted to different cultural settings, including those from LMICs [35]. A recent systematic review found that studies evaluating culturally tailored, evidence-based interventions for people with dementia—while maintaining core therapeutic components, such as cognitive stimulation—were acceptable, feasible, and effective in certain minority groups and LMICs, with outcomes comparable to those of the original interventions [36]. Beyond the health sector, social prescribing seeks to improve access of people living with dementia and their caregivers to non-clinical support and services offered in the community to address their different needs and reduce social isolation [37]. While still in its early stages in countries such as Nigeria, The Philippines and Indonesia, its focus on acting on health determinants, identifying and reaching deprived communities and addressing inequalities offers significant opportunities [38]. To materialize these benefits, considering the existing limitations in LMICs, a social prescribing implementation must be context-sensitive, co-designed with local stakeholders to identify barriers and enablers, asset-based, leveraging existing community infrastructure, and highly coordinated with clear referral pathways [38].

At the global level, there is growing recognition of the importance of developing national strategies to address dementia. The WHO Global Action Plan (GAP) for Dementia has urged member states to make dementia a public health priority by creating National Dementia Plans, focusing on six key areas: awareness and friendliness, risk reduction, diagnosis and care, caregiver support, information systems, and research and innovation [39]. However, by May 2024, 48 countries had achieved this goal, and only two were LMICs [40]. After the active work of various key organisations, a six-year extension has been granted to keep working towards achieving the GAP’s targets given the importance of taking measures to prioritise brain health [41]. Yet, National Dementia Plans will only achieve their goals if they are adequately resourced and implemented.

In addition, efforts to improve the representativeness of dementia research are making headway [42]. For example, the African Dementia Consortium aims to generate genetic, clinical, and socioeconomic data from African populations [43]. The Latin America and the Caribbean Consortium on Dementia (LAC-CD) is another initiative promoting research activities to identify the unique genetic, social, and economic factors that drive Alzheimer’s and frontotemporal dementia presentation in LAC [44]. Also, the fourth phase of the Alzheimer’s Disease Neuroimaging Initiative (ADNI4) seeks to increase the generalisability of its findings by including more participants from underrepresented populations [45].

### Towards transforming dementia care in LMICs

Key areas for transformation include embracing a culture of high-quality health systems beyond merely reacting to disease and disease management, strengthening primary care systems in the true sense with active and robust community engagement, integrating dementia care with other chronic disease management programs, and ensuring that healthcare professionals receive training in dementia diagnosis and care. Governments, international organisations, and civil society must work together to create and implement policies that protect the rights of people living with dementia and support families who provide care. Additionally, community-based programs focusing on awareness-raising, stigma reduction, and caregiver support can be pivotal in changing the landscape of dementia care in LMICs.

To move forward, we must develop innovative financing models to fund healthcare and social care for people with dementia and their caregivers, expand local research efforts to generate data on dementia prevalence and risk factors and establish partnerships to scale and sustain successful interventions. While specific policy models for dementia care remain underdeveloped globally—particularly in LMICs—this article invites a rethinking of conventional approaches, recognising that current global shifts in health financing offer a timely opportunity to innovate beyond high-income country templates.

### Leapfrogging the response to dementia in LMICs

The concept of leapfrogging, championed by the World Economic Forum, emphasises the opportunity for LMICs to bypass traditional pathways of development used by HICs and move directly toward more sustainable and equitable health systems [46]. This approach leverages the structural conditions and emerging technologies in LMICs to avoid replicating the inefficiencies and inequities seen in HICs.

The risk of following the same pathways as HICs, beyond the prohibitive costs for many LMICs and their reliance on hospital-centric approaches, lies in perpetuating health disparities, such as the underrepresentation of minority racial and ethnic groups and socioeconomic inequities. In HICs, dementia research has largely focused on participants from Caucasian or white populations, neglecting other communities. Additionally, the traditional top-down approach to research prioritisation often leads to a gap between scientific investigation and the real-world needs of people living with dementia. LMICs have the opportunity to learn from these lessons and avoid major pitfalls linked to specialist-centred healthcare structures by embracing innovative approaches that reflect their unique contexts, cultures, and resources. By leapfrogging, moving away from “copy and paste” healthcare delivery methods, LMICs can build more inclusive, adaptable, and efficient health systems that better serve their populations.

### Dementia as a tracer condition to benchmark health system performance

Dementia can serve as a tracer condition—a focal point through which health systems can identify weaknesses, generate insights, and develop broader solutions applicable to a range of health challenges [39,47]. Unlike other health conditions, dementia exposes critical gaps in healthcare delivery, including the complex interaction between cognitive decline, multimorbidity, carer burden, and societal awareness. By working to improve dementia care, LMICs can simultaneously uncover opportunities to strengthen their overall health systems, particularly in managing chronic conditions and multimorbidity.

Using dementia as a tracer condition allows for a more comprehensive understanding of the healthcare ecosystem. It highlights not only the need for medical treatment but also the broader social and community-based supports required to manage long-term, complex conditions. This approach challenges the traditional view that health systems are limited to healthcare delivery and underscores the essential role that community engagement, health literacy, and cross-sector collaboration play in promoting better health outcomes.

By focusing on dementia as a tracer condition, LMICs can develop scalable, evidence-based interventions that not only improve dementia care but also serve as models for addressing other chronic diseases. For instance, in Brazil, cognitive stimulation therapy (CST), was adapted through a collaborative process involving health professionals from different disciplines and caregivers. Their input highlighted key contextual factors that needed to be addressed for successful implementation, such as the lack of secure transportation for PWD, the need to increase caregiver knowledge about CST and its benefits using awareness and informational campaigns, as well as the need to adjust contents to include culturally familiar activities and information that reflect the Brazilian reality [48]. A randomized controlled trial of the adapted version demonstrated its feasibility, showing high acceptance among participants and improved mood for PWD [49].

Another example is the LatAm-FINGERS study, an initiative that is currently conducting the first non-pharmacological clinical trial involving participants from 12 Latin American countries to evaluate a lifestyle multidomain intervention previously implemented in HICs. The study has employed innovative and culturally sensitive recruitment methods, such as respecting local culture, leveraging the knowledge of local stakeholders and implementing awareness campaigns [50]. To promote participant engagement, their strategies have included moderated chat groups, dietary adaptations to local habits, shared cultural celebrations, personalised video tutorials, engagement calls, among others [51].

The lessons learned from dementia care—such as the integration of services, the importance of caregiver support, and the need for cross-sector collaboration—can be applied to a wide range of health conditions, leading to improvements in both the efficiency and equity of health systems [52]. Using dementia as a tracer condition can drive societal, scientific and health communities towards high-quality health systems that measure and report what matters most to people, such as competent care, user experience, health outcomes, and confidence in the system [53].

## The Peruvian case

Peru is a middle-income country in South America with vast geographical and cultural diversity and significant inequalities. Its health system is highly fragmented and complex [54], with healthcare provision and funding managed by multiple entities, both public and private, under the oversight of the Ministry of Health (MINSA). The two main public health providers are MINSA and the Social Security System (EsSalud).

### Dementia diagnosis and care challenges in Peru

Dementia is challenging to diagnose in Peru due to low public awareness, limited healthcare access, and a lack of appropriate training among health professionals [55]. Illiteracy rates among adults over 60 vary significantly, with 9% in urban areas and 34% in rural areas [56], making many cognitive assessment tools developed in HICs unsuitable for direct use [57]. Social services, such as skilled nursing facilities, are scarce and often accessible only to wealthier individuals, while most caregiving falls on family members, often untrained and unsupported financially. This situation is mirrored in other Latin American countries, such as Brazil and Argentina, where dementia specialists are also in short supply, and health systems struggle to support dementia care across diverse populations [58,59].

### Case study: Emerging opportunities in Peru

Peru has started addressing these issues through several recent legislative reforms. In a significant step forward, the 2018 Law for the Prevention and Treatment of Alzheimer’s Disease and Other Dementias (Law 30795) established a more comprehensive legal framework that includes directives for prevention, assessment, diagnosis and the promotion of integrated healthcare to protect and support individuals living with dementia [60]. Moreover, the health ministry, non-profit organisations and Alzheimer’s Disease International (ADI) have engaged in discussions to promote the development of a National Dementia Plan. Taken together, these policy actions provide a critical window of opportunity to position dementia as a government priority, establish minimum standards of care and guide a more equitable and efficient resource allocation to meet the growing demand for health services [61].

An ongoing mental health reform also represents a significant opportunity for dementia care as it emphasises community-based approaches supported by a growing network of Community Mental Health Centres [62]. Also, a National Observatory for Alzheimer’s disease and other dementias was launched in 2024, marking a significant step toward greater awareness and more transparent case registration [63]. However, ensuring clarity on the data collection process and its quality remains essential to making this observatory a truly valuable tool in the dementia response.

While these reforms are promising, they are not yet backed by sufficient budgets to ensure full implementation [55]. This situation is not unique to Peru—many LMICs introduce progressive legislation or reforms without adequately resourcing them. However, the emphasis on community-based care and task-shifting through training non-specialists, as seen in Peru, holds promise as a model that can be adapted elsewhere.

### Digital health advances

Peru’s strides toward a digital government have extended into healthcare, with the government supporting telehealth services and piloting electronic clinical records in hospitals and primary care [63]. This embrace of digital health creates a promising base for adopting low-cost tools for dementia risk assessment and cognitive screening that are already being used for dementia diagnosis in other Latin American countries [58]. While the COVID-19 pandemic accelerated the routine use of digital tools, gaps remain in scaling these innovations effectively. Other LMICs have similarly adopted telemedicine and digital health solutions, particularly post-pandemic, yet face challenges in sustaining these efforts and integrating them within fragmented health systems [59]. Learning from these examples, Peru can contribute to global conversations on using digital tools to support underserved populations, particularly in dementia care.

### Using research to foster change

The “Innovations using mHealth for People with Dementia and Co-Morbidity and their Carers” (IMPACT Salud) is a four-year project that started in 2023 and is designed to strengthen health systems using dementia as a tracer condition through sustainable, community-delivered, technology-enabled innovations in Peru [64,65].

One of the project’s work streams focuses on evaluating the capacity of the Peruvian health system to provide diagnosis and care for dementia, while also documenting the experiences of patients and their caregivers [66]. To our knowledge, this assessment has generated, for the first time, systematic evidence on the complexities of healthcare provision for dementia in Peru, filling a critical knowledge gap and enabling the identification of key stakeholders who could drive health sector reforms. Moreover, these findings are also informing evidence-based adaptations of an intervention, led by another work stream of the project, which will provide practical guidance and tools to enhance the quality of life of individuals living with dementia, as well as to support the mental well-being and overall quality of life of their caregivers.

Another key strategy is to use research as an opportunity to raise public awareness of dementia and reduce stigma. Beyond building connections and partnerships with stakeholders from the health system, government, and academic institutions, particular emphasis has been placed on engaging the broader community through public events such as a brain health awareness marathon [67,68]. In addition, well-established communication channels, including local radio programs and social media platforms have been used to disseminate information and foster dialogue about dementia. Furthermore, a Lived Experience Advisory Panel with caregivers has been formally established to create spaces for collaboration and co-design, enabling the exchange of ideas, a deeper understanding of needs, and the joint development of proposals.

IMPACT Salud is also exploring how to use technology to reach more potential patients and increase diagnosis rates within the Peruvian context. They are currently testing a diagnostic screening mHealth system, which was co-designed with community members and community health workers to ensure adaptability and efficacy [69,70]. In addition, the project plans to use the collected data to evaluate accuracy of the diagnostic tools and to generate evidence on dementia-related health resource usage and costs in the Peruvian population.

The project is still ongoing, and while the results of these strategies are still being collected, preliminary findings indicate promising progress.

## Conclusion

Cognitive decline presents challenges across multiple sectors of society, both within and beyond health systems. Transformations in the response to dementia are essential to improve the quality of life and promote the dignity and autonomy of people living with this condition. New approaches must reduce health disparities, combat stigma and discrimination, and foster a more inclusive, equitable, and supportive society.

To address the complex challenges dementia poses in LMICs while using it as an opportunity to strengthen health systems, a paradigm shift is essential. Instead of importing or replicating solutions from HICs, LMICs need to focus on leapfrogging the mistakes made elsewhere by developing context-specific responses. This requires a nuanced understanding of the local challenges, resources, and sociocultural contexts.

Governments should prioritise the creation and implementation of adequate funded comprehensive national dementia plans that address prevention, diagnosis, care, and support for caregivers. Emphasising community-based care models that leverage local resources and provide training for non-specialist health workers to deliver dementia care is crucial. Public awareness campaigns should be implemented to reduce stigma and improve understanding of dementia, encouraging early diagnosis and intervention. Investments in strengthening health systems to provide integrated care for dementia and other chronic conditions are essential, ensuring coordination between different levels of care. Developing programs to support family caregivers, including financial assistance, training, and respite care services (e.g., day care services), will help alleviate the burden on families.

While existing evidence and interventions can provide a starting point, they are just one piece of a larger puzzle. By aligning these interventions with the realities of each unique context, LMICs can craft tailored responses that genuinely meet the needs of those affected by dementia. Task-shifting, digital health innovations and participatory community-driven approaches represent ways forward for improving dementia care and, by extension, strengthening the broader health system. This approach has the potential to inform not only national dementia strategies but also health systems strengthening across LMICs, enabling them to avoid pitfalls experienced by HICs.

Accepting that innovation may come with the possibility of failure, health systems in LMICs can move towards genuinely context-specific, sustainable progress. As Abimbola succinctly put it, “We interpret our realities and the systems within which we have our being.” [71]. This is not about innovation for its own sake but about crafting effective, inclusive responses adapted to the real needs of people facing dementia across diverse global settings.

## Supporting information

S1 Fig Leapfrogging dementia care in LMICs. Figure created by the authors with assistance of AI tool Napkin AI. (TIFF)
