# Supplementary figures and images for: Rethinking and transforming health systems for dementia care in low- and middle-income country settings
Source: PLOS Glob Public Health. 2025 Dec 3;5(12):e0005419. doi: 10.1371/journal.pgph.0005419 (PMC12674554; doi:10.1371/journal.pgph.0005419)

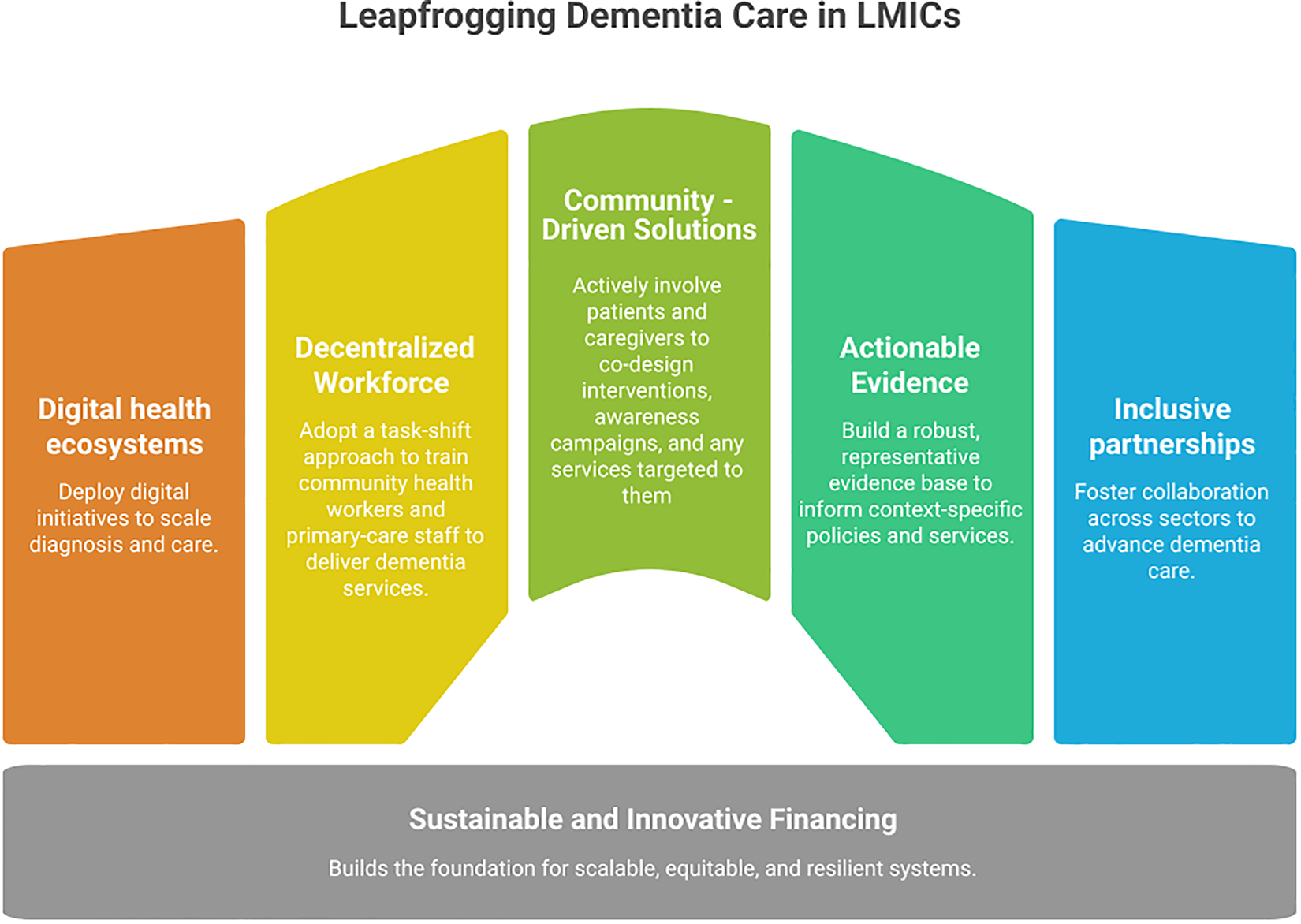

Supplement: S1 Fig — (TIFF) [file pgph.0005419.s001.tif]
